# Integrative In Silico and In Vivo Evidence of Quercetin as a Multitarget Neuroprotective Agent in Alzheimer’s Disease

**DOI:** 10.1021/acsomega.6c00778

**Published:** 2026-04-29

**Authors:** Thiago Malverde de Oliveira, Lucas Diego Pereira Bento, Isabela Santos de Melo Wiermann, Bianca de Souza Fonseca, Guilherme Saraiva Tsui, Carolina Jayne Pereira de Jesus, Raphaela Oliveira Sales, Mateus Antonio Pereira Prado, Mateus Silva de Castro, João Pedro Reis Moura, Ellen Nunes Gomes, Lavinia Brito Bastos, Izabela Cristina Lima Orsine, Lucas de Souza Esteves, Maria Clara Silva Soares, Daniel Luciano Falkoski, Michel Pires da Silva, Tiago Alves de Oliveira, Alisson Marques da Silva, Eduardo Habib Bechelane Maia, Franco Henrique Andrade Leite, Marcelo Siqueira Valle, Paulo Batista de Carvalho, Liliane Costa Vanessa Pereira Mendes, Alex Gutterres Taranto, Laila Cristina Moreira Damázio

**Affiliations:** † Department of Natural Sciences, Federal University of São João del-Rei, Dom Helvécio Square, 74-Dom Bosco, São João del-Rei, Minas Gerais 36301-160, Brazil; ‡ Department of Biotechnology, Federal University of São João del-Rei, Dom Helvécio Square, 74-Dom Bosco, São João del-Rei, Minas Gerais 36301-160, Brazil; § Department of Medicine, Federal University of São João del-Rei, Dom Helvécio Square, 74-Dom Bosco São João del-Rei, Minas Gerais 36301-160, Brazil; ∥ Department of Computer Science, State Federal Center for Technological Education of Minas Gerais, R. Alvares de Azevedo, Divinópolis, Minas Gerais 35503-822, Brazil; ⊥ Department of Health Science, State University of Feira de Santana, Transnordestina Avenue, Feira de Santana, Bahia 44036-900, Brazil; # Feik School of Pharmacy, 130378 University of the Incarnate Word, San Antonio, Texas 78209, United States

## Abstract

Alzheimer’s disease requires therapeutic strategies targeting multiple pathological mechanisms. This study investigates the neuroprotective potential of quercetin using an integrated approach combining in silico modeling and in vivo validation. Computational analyses examined the binding behavior of quercetin toward three key enzymes implicated in disease pathophysiology: acetylcholinesterase, butyrylcholinesterase, and beta-secretase 1. Molecular dynamics simulations reveal consistent interaction patterns and stable binding profiles across independent trajectories. For experimental validation, Wistar rats with surgically induced Alzheimer’s disease were orally treated with quercetin (30 mg/kg) for 5 weeks. Histological and immunohistochemical analyses of the hippocampus and subventricular zone evaluated neuronal density and astrocytic activation using glial fibrillary acidic protein and vimentin markers. Computational results supported the multitarget potential of quercetin through stable enzyme interactions. Consistently, in vivo assays demonstrated increased neuronal density and reduced astrocytic marker expression, suggesting a protective modulation of neuroinflammatory processes. These findings highlight quercetin as a promising scaffold for multitarget therapeutic strategies aimed at mitigating the progression of Alzheimer’s disease.

## Graphical abstract

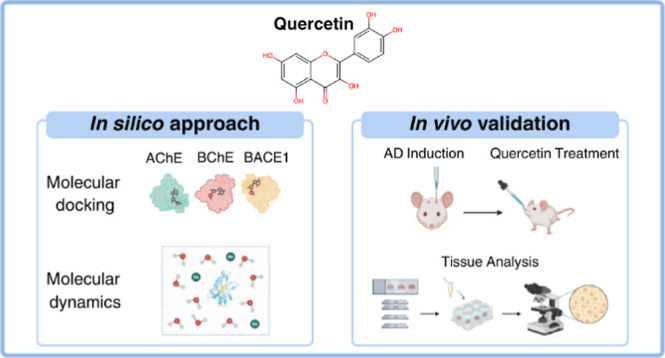

## Introduction

Alzheimer’s disease (AD) is a progressive, multifactorial neurodegenerative disorder and the leading cause of dementia worldwide. It is characterized by cognitive decline, memory impairment, and behavioral alterations, affecting over 55 million individuals globally, a number projected to rise sharply in the coming decades.^1^ Neuropathologically, AD is defined by the accumulation of amyloid-beta (Aβ) plaques, neurofibrillary tangles formed by hyperphosphorylated tau proteins, oxidative stress, synaptic dysfunction, and neuronal loss.^2^

Cholinergic deficits represent a hallmark of AD and are closely associated with Aβ pathology. Reduced acetylcholinesterase (AChE) activity, coupled with increased butyrylcholinesterase (BChE) activity, particularly in the hippocampus, contributes to episodic memory impairment.^3^ Acetylcholine, essential for learning, memory, and attention, is synthesized by choline acetyltransferase from choline, acetyl-CoA, and ATP. In AD, reduced enzymatic activity in the cortex, hippocampus, and amygdala results in diminished synaptic acetylcholine, an early pathogenic hallmark. Consequently, AChE inhibitors remain the mainstay of current symptomatic therapy.^4^

The amyloid cascade hypothesis proposes that the amyloid precursor protein (APP) triggers Aβ production, aggregation, and neurotoxicity.^5^ Beta-secretase 1 (BACE1) plays a pivotal role in this process, initiating APP cleavage and the formation of neurotoxic Aβ peptides. The Aβ_1–42_ [Fn fn1] species, highly prone to aggregation, accumulates across several brain regions, including the cortex, hippocampus, amygdala, frontal lobe, cingulate gyrus, substantia nigra, and brainstem nuclei, leading to synaptic dysfunction, neuronal loss, and cognitive decline.^6^ Concurrently, tau hyperphosphorylation destabilizes microtubules and promotes neurofibrillary tangle formation, further exacerbating neuronal dysfunction and death.^7^ Together, these mechanisms highlight the multifactorial nature of AD and the necessity for therapeutic strategies targeting multiple pathological pathways.

Beyond oxidative stress, several natural polyphenols have been reported to directly modulate amyloidogenic pathways involved in AD. In particular, several flavonoids have demonstrated the ability to interfere with Aβ aggregation processes, including inhibition of fibril formation and destabilization of preformed aggregates, thereby attenuating Aβ-induced neurotoxicity.^8,9^ Among natural compounds with multitarget potential, flavonoids have attracted increasing attention as candidate therapeutics due to their antioxidant, anti-inflammatory, and neuroprotective properties.^10^ Quercetin (2-(3,4-dihydroxyphenyl)-3,5,7-trihydroxychromen-4-one), abundant in fruits, vegetables, and tea, possesses a polyphenolic scaffold that confers potent antioxidant capacity.^11^

The integration of virtual high-throughput screening (vHTS) workflows with artificial intelligence (AI)-driven modeling strategies has emerged as a powerful approach for the rational discovery of multitarget modulators, enabling the exploration of complex polypharmacological profiles.^12,13^ In this context, the BioMolExplorer computational workflow was designed to address the challenges of assembling high-quality data sets for target-focused drug discovery.^14^ By integrating and standardizing information from established repositories such as the Protein Data Bank (PDB),^15^ ChEMBL,^16^ and ZINC,^17^ the tool retrieves bioactive molecules, structurally related analogs, and relevant protein–ligand complexes associated with predefined therapeutic targets. Its built-in filtering and automated redocking curation of protein structures ensure the generation of consistent, research-ready data sets that support downstream computational analyses, including the molecular docking and molecular dynamics (MD) simulations employed in this study, facilitating the investigation of multitarget agents such as quercetin and its interactions with AChE, BChE, and BACE1.

To validate the computational predictions, an in vivo experimental model of sporadic AD was established via intracerebroventricular (ICV) administration of streptozotocin (STZ), a well-characterized approach that reproduces key neuropathological and cognitive features of the disease, including oxidative stress, neuronal loss, and astroglial activation.^18^ Histological analysis using Nissl staining assessed neuronal integrity in hippocampal regions,^19^ while immunohistochemistry for glial fibrillary acidic protein (GFAP) and vimentin provided insights into astrocytic reactivity and neuroinflammatory responses.^20^ This experimental validation complements the computational phase, establishing a translational framework that bridges in silico predictions with in vivo neuroprotective evidence.

Given the urgent need for more effective therapeutic strategies against AD, this study integrates computational and experimental approaches to identify multitarget inhibitors targeting key enzymes involved in AD pathology: AChE, BChE, and BACE1. Using in silico vHTS combined with AI, candidate compounds were prioritized for experimental validation. Their neuroprotective potential was subsequently evaluated in Wistar rats with STZ-induced AD, focusing on neuronal integrity and astrocytic reactivity. This integrative and polypharmacological framework aims to identify compounds that simultaneously modulate multiple pathological pathways, thereby offering a more effective and comprehensive strategy to mitigate or delay AD progression.

## Results and Discussion

This study was motivated by the need to establish a direct link between molecular-level interactions and biologically measurable neuroprotective outcomes using an integrated chemical-biological framework. By combining AI-assisted vHTS, structure-based computational modeling, and in vivo histological evaluation, the present work bridges chemical scaffold selection, target-specific binding behavior, and functional responses in the central nervous system (CNS). This multidisciplinary strategy enables the interpretation of computational predictions within a biological context, allowing the assessment of quercetin not only as a bioactive flavonoid but as a chemically defined multitarget scaffold whose interaction profile translates into neuronal preservation and modulation of astroglial responses.

The vHTS-AI BioMolExplorer tool successfully identified quercetin as the lead natural compound. Quercetin (Figure 1) is a flavonoid well recognized for its antioxidant and anti-inflammatory properties and has been extensively investigated for its neuroprotective effects.^21^ Previous in vitro studies have shown that quercetin attenuates pro-inflammatory cytokine expression, protects hippocampal neurons from oxidative stress, modulates synaptic plasticity, and inhibits key enzymes such as AChE and BACE1.^22^ Consistently, combined in silico and in vivo analyses demonstrated that quercetin establishes stable molecular interactions with enzymes implicated in disease pathophysiology and promotes increased neuronal density together with reduced astrocytic marker expression, reflecting robust neuroprotective effects mediated by the modulation of neuroinflammation and targeted engagement of CNS pathways.^21^

**Figure 1:**
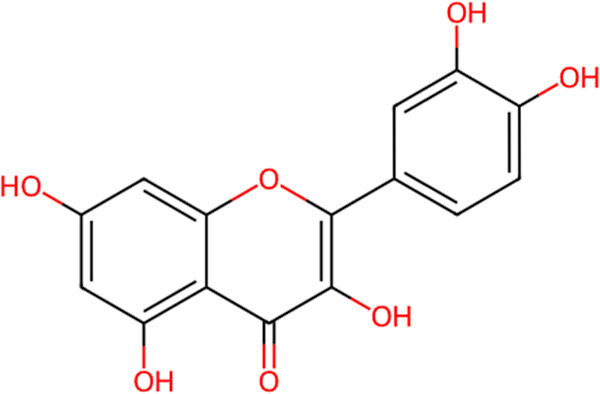
Chemical structure of quercetin obtained from the ChEMBL database (ChEMBL ID: CHEMBL50). Functional groups highlighted in red correspond to phenolic hydroxyl groups (–OH), the carbonyl group (C=O), and oxygen atoms of the heterocyclic ring, which are associated with antioxidant activity and potential intermolecular interactions.

### Computational In Silico Model

The in silico evaluation began with the definition of chemical exploration domains for AChE (ID: CHEMBL220), BChE (ID: CHEMBL1914), and BACE1 (ID: CHEMBL4822). Only entries derived from *Homo sapiens*, classified as “single protein,” and matching the nomenclature of these enzymes were included.

The ChEMBL Python library was used to retrieve bioactive compounds and structurally related analogs for each target based on these identifiers. Flavonoids were prioritized as a subgroup of interest among the bioactives, and only small molecules with *K*_i_ values ≤1000 μM were retained. The selection of structurally related compounds followed the same prioritization, applying additional filters: at least 70% structural similarity to the bioactives, classification as a small molecule, and molecular weight ≤500 Da. The resulting compound sets are summarized in Table 1.

**Table 1:** Compounds Retrieved from the ChEMBL Database for Each Explored Enzymatic Identifier

ChEMBL	bioactives	related structures	total
AChE	535	169	704
BChE	290	84	374
BACE1	1159	78	1237

Once the relevant data sets were retrieved (Table 1), Venn diagrams were generated to identify molecular subsets shared among the target enzymes. This approach aimed to uncover structural intersections that could highlight central scaffolds shared among them and potentially reinforce their capacity for multitarget interactions. Figure 2 depicts the resulting clusters and overlaps identified among the retrieved compounds.

**Figure 2:**
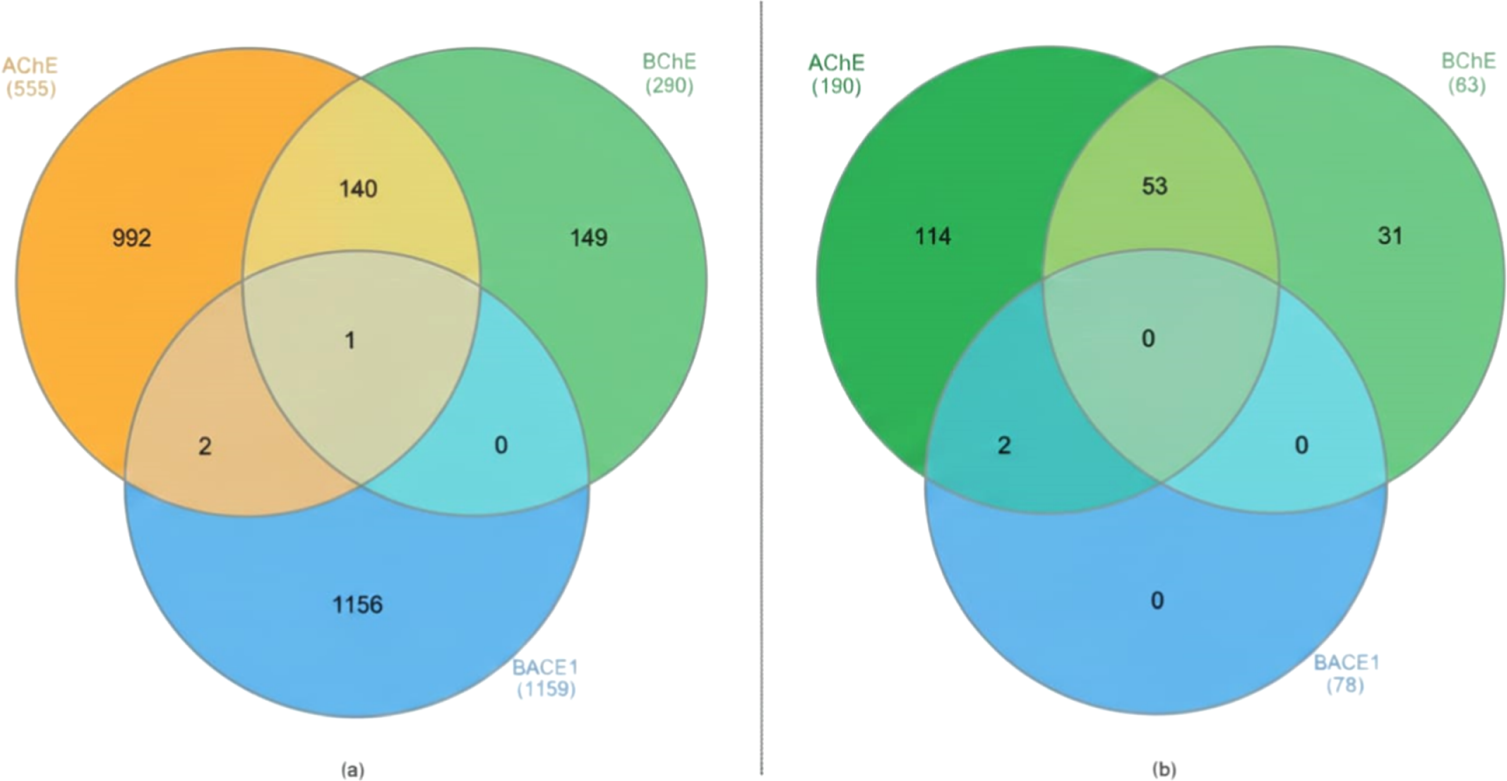
Venn diagrams showing the overlap of compounds associated with AChE, BChE, and BACE1. (a) Distribution of 1984 bioactive compounds retrieved from ChEMBL based on *K*_i_ ≤ 1000 μM. (b) Structural analogs (*n* = 331) selected by ≥70% similarity and molecular weight ≤500 Da, highlighting subsets with potential multitarget relevance.

As illustrated in Figure 2, substantial overlaps are observed between compounds associated with the cholinesterases AChE and BChE, reflecting a strong structural correlation between these enzymes. The active site of cholinesterases consists of a deep catalytic gorge with two principal regions: the catalytic anionic site (CAS) and the peripheral anionic site (PAS).^23^ The PAS, positioned at the entrance of the catalytic cleft, is enriched in aromatic residues such as tryptophan and tyrosine, which play essential roles in ligand recognition and stabilization.

In contrast, the overlap involving BACE1 is markedly restricted. Only two compounds, CHEMBL464006 and CHEMBL518543 (Figure 3), corresponding to the flavonoids *murisin* and *kuwanon C*, were found to be shared between AChE and BACE1. Both display highly functionalized polycyclic frameworks with multiple fused rings and extensive substitution. This architectural complexity suggests that cross-target affinity between cholinesterases and BACE1 may require elaborate molecular frameworks capable of engaging structurally distinct catalytic regions that are far less conserved among these enzymes. Such evidence indicates that dual-target activity requires not only steric compatibility but also a refined balance of hydrophobic and electronic properties.

**Figure 3:**
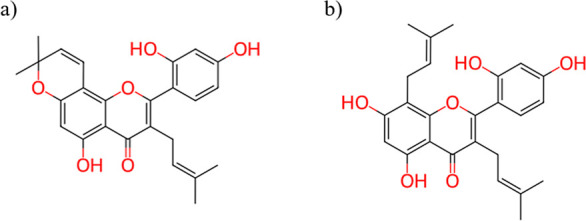
Molecular structures of (a) *morusin* (CHEMBL464006) and (b) *kuwanon C* (CHEMBL518543), the only two compounds simultaneously shared by AChE and BACE1 in the structural overlap analysis. Functional groups highlighted in red correspond to phenolic hydroxyl groups and oxygen atoms of the flavonoid core that may contribute to hydrogen-bond interactions and antioxidant properties.

Within this structural landscape, quercetin stands out as a compelling multitarget candidate. Its planar polyphenolic scaffold and multiple hydroxyl groups enable π–π stacking with PAS aromatic residues and hydrogen bonding with the polar side chains of cholinesterases. At the same time, its extended conjugation and functional diversity provide the flexibility required for productive interactions with BACE1. These molecular features, combined with its favorable physicochemical properties, including a molecular weight of 302.24 g/mol, approximately 22 heavy atoms, seven hydrogen bond donors, and compliance with Lipinski’s rules,^24^ suggest drug-like characteristics relevant for central nervous system drug discovery, although the bioavailability and blood–brain barrier permeability of quercetin are known to be limited due to rapid metabolism.^9^

Redocking analysis enabled the selection of crystallographic structures with the lowest root-mean-square deviation (rmsd) values for each target, yielding the complexes 4M0E for AChE (0.15 Å), 1P0I for BChE (1.77 Å), and 5HE5 for BACE1 (0.17 Å) for subsequent computational evaluation. Molecular docking analyses revealed favorable intermolecular interactions between quercetin and all selected enzymatic targets, as assessed using AutoDock Vina^25^ and DOCK6.^26^

The resulting docking scores suggested favorable interactions with all evaluated enzymes, with subtle variations among the targets. Because the scoring functions implemented in AutoDock Vina^25^ and DOCK6 ^26^ operate on different energy scales, the reported values should be interpreted as relative ranking metrics rather than physical binding free energies (Δ*G*). Among the evaluated targets, BChE exhibited the most favorable score, followed by AChE and BACE1, as summarized in Table 2. The consensus score was used as a comparative ranking metric integrating the results obtained from both docking programs.

**Table 2:** Docking Scores for Quercetin against the Target Enzymes^a^

target	AutoDock Vina	DOCK6	consensus
AChE	–8.37	–40.05	–24.21
BChE	–9.36	–40.90	–25.13
BACE1	–8.02	–40.71	–24.37

a The consensus score represents a comparative ranking metric derived from both docking methods.

Based on the interactions identified by molecular docking, a detailed residue-level assessment of quercetin binding at each active site was performed using Discovery Studio Visualizer.^27^ In addition, footprint analysis from DOCK6 ^26^ was used to quantify and map the individual energetic contributions of each residue. For each enzyme, the 50 residues with the highest total energy contributions were analyzed, enabling the identification of the principal structural determinants underlying complex stability. The results are presented in Figure 4, highlighting specific interaction patterns and the recurrence of key residues across the targets.

**Figure 4:**
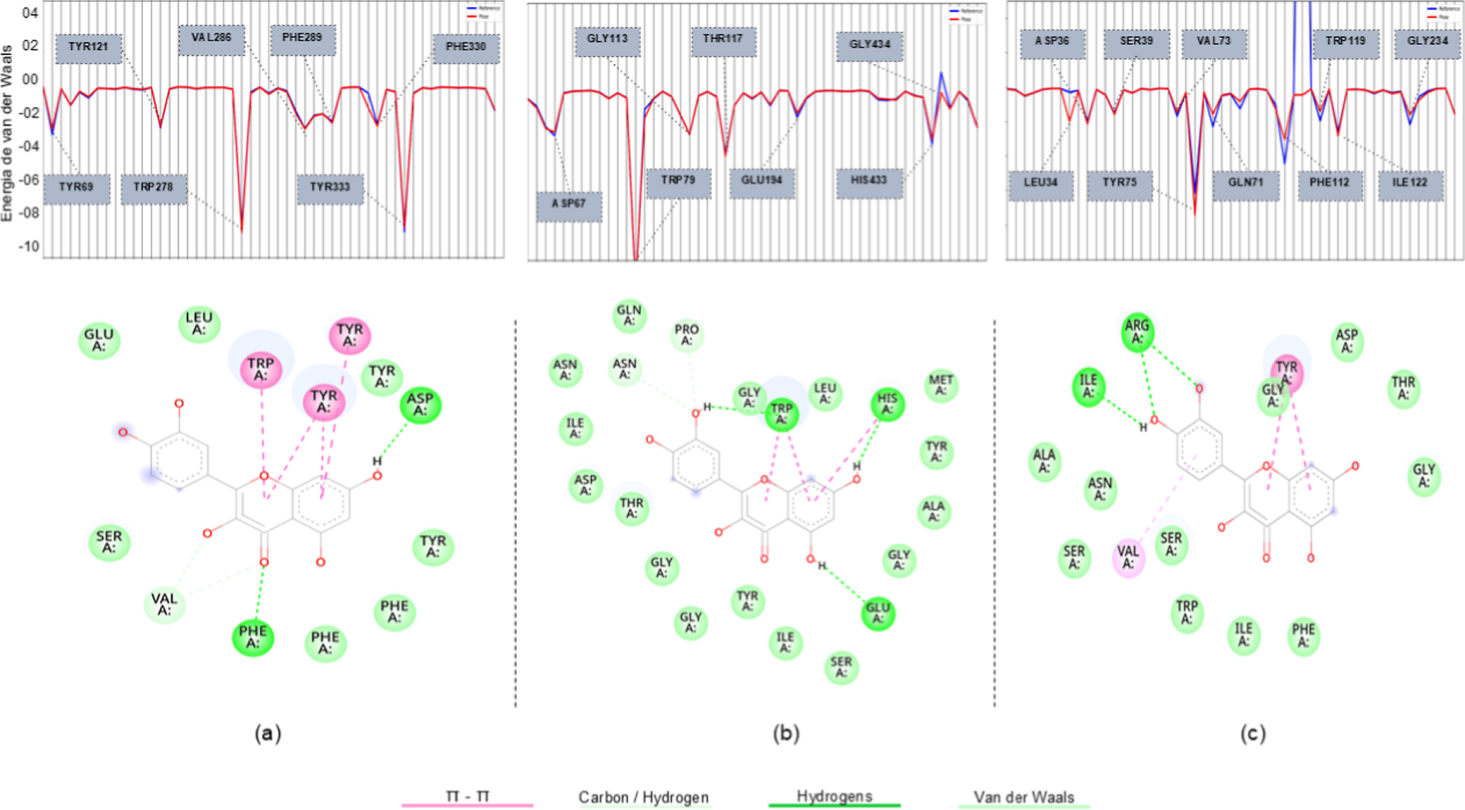
Energy profiles and molecular interaction diagrams for quercetin with (a) AChE, (b) BChE, and (c) BACE1. The upper panels depict van der Waals energy contributions per residue, while the lower panels illustrate specific interactions, including π–π stacking, carbon–hydrogen contacts, conventional hydrogen bonds, and van der Waals interactions.

As shown in the AChE binding site (Figure 4a), a key enzyme in neurotransmission, quercetin interacts with residues located in both the CAS and PAS. The Trp278 and Phe330 amino acids contribute prominently through van der Waals interactions, guiding ligand orientation toward the catalytic region and suggesting possible modulation of enzymatic activity. Tyr333 further stabilizes the complex through hydrogen bonding and π–π stacking. Peripheral residues Tyr121 and Tyr69, situated in the PAS, are essential for substrate entry, exit, and allosteric modulation; interactions with these residues suggest that quercetin may influence AChE activity by altering substrate accessibility or inducing conformational adjustments.

In BChE (Figure 4b), the interaction profile mirrors what was observed for AChE, with aromatic residues playing a central role in docking quercetin. Among these, Trp79 provides substantial hydrophobic stabilization. Hydrophobic carbon–hydrogen contacts involving Gly113, Gly434, and His433 further reinforce ligand binding, reflecting the greater conformational flexibility and distinct spatial organization of the BChE active site relative to AChE. Together, these residues support a favorable microenvironment in which nonpolar, aromatic, and polar interactions collectively stabilize the complex.

For BACE1 (Figure 4c), the persistent involvement of aromatic residues such as Tyr75 and Trp191 highlights the importance of π–π interactions and hydrogen bonding in docking polyphenolic ligands. A direct hydrogen bond to Asp36, a negatively charged catalytic residue, indicates a specific electrostatic environment suited for binding hydroxylated compounds such as quercetin. Hydrogen bonding involving Tyr75 and Asp36 is particularly important for proper ligand orientation and recognition, potentially enabling interaction with the enzyme’s flap region, which regulates access to the active site and stabilizes inhibitory complexes. Additional hydrophobic interactions with Trp191 and Gly34 further enhance complex stability, with Gly34 positioned near the flap, facilitating optimal ligand accommodation within this functionally significant region.

MD simulations provided detailed insight into the conformational behavior of the quercetin-enzyme complexes (Figures 5–7). Root-mean-square deviation (rmsd), root-mean-square fluctuation (rmsf), radius of gyration (Rg), and protein–ligand hydrogen bonds were monitored over 50 ns trajectories to evaluate structural stability under physiologically relevant conditions. To assess the robustness of these observations, additional independent replicate simulations were performed using different initial velocity seeds (Figures S1–S6). Although individual trajectories exhibited some variability, consistent dynamic patterns were identified across replicates.

**Figure 5:**
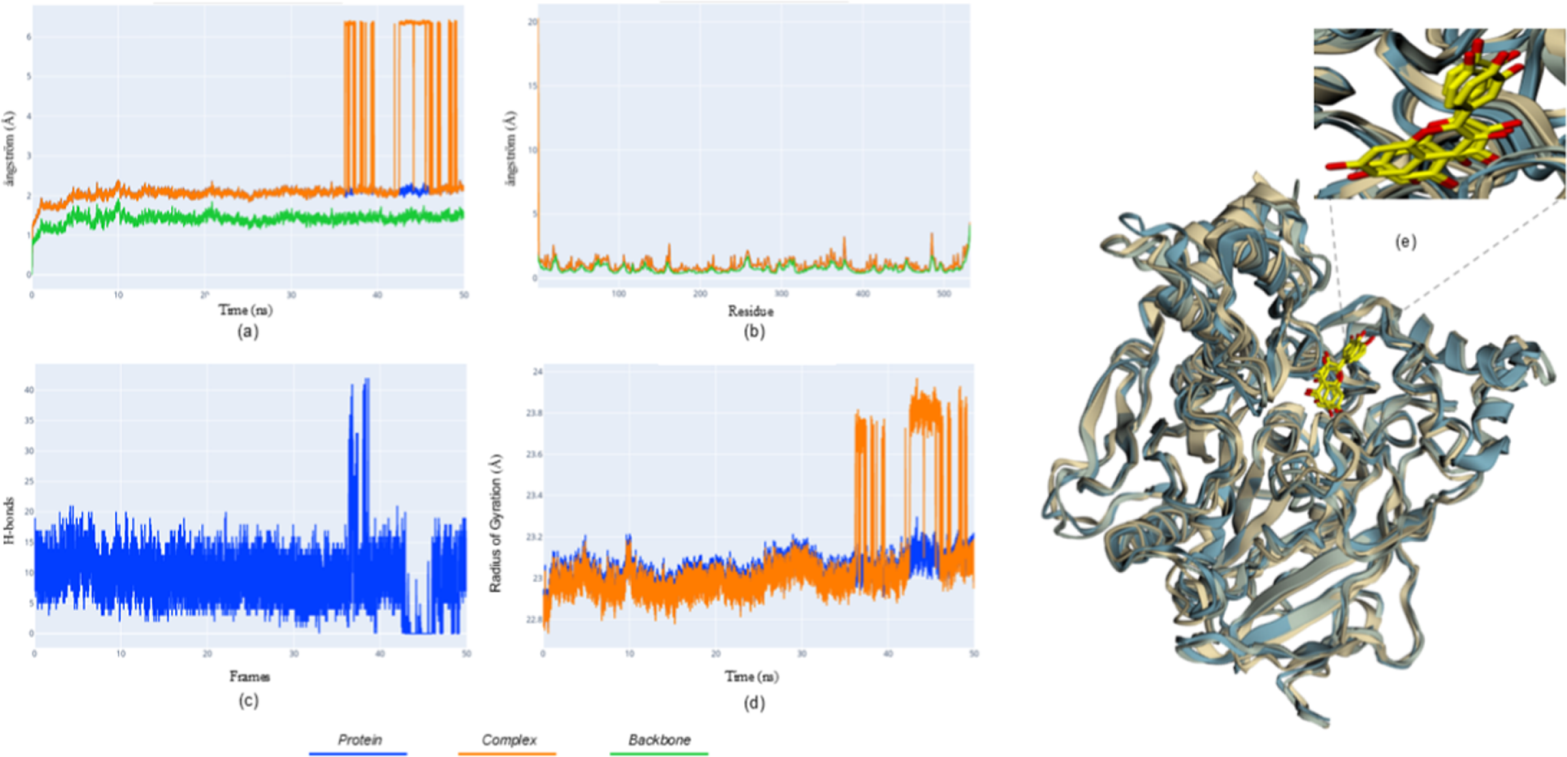
Molecular dynamics results for the AChE-quercetin complex. (a) rmsd; (b) rmsf; (c) hydrogen bonds; (d) Rg for the isolated protein (blue), complex (orange), and backbone (green); (e) superposed frames highlighting ligand movement toward the PAS.

**Figure 6:**
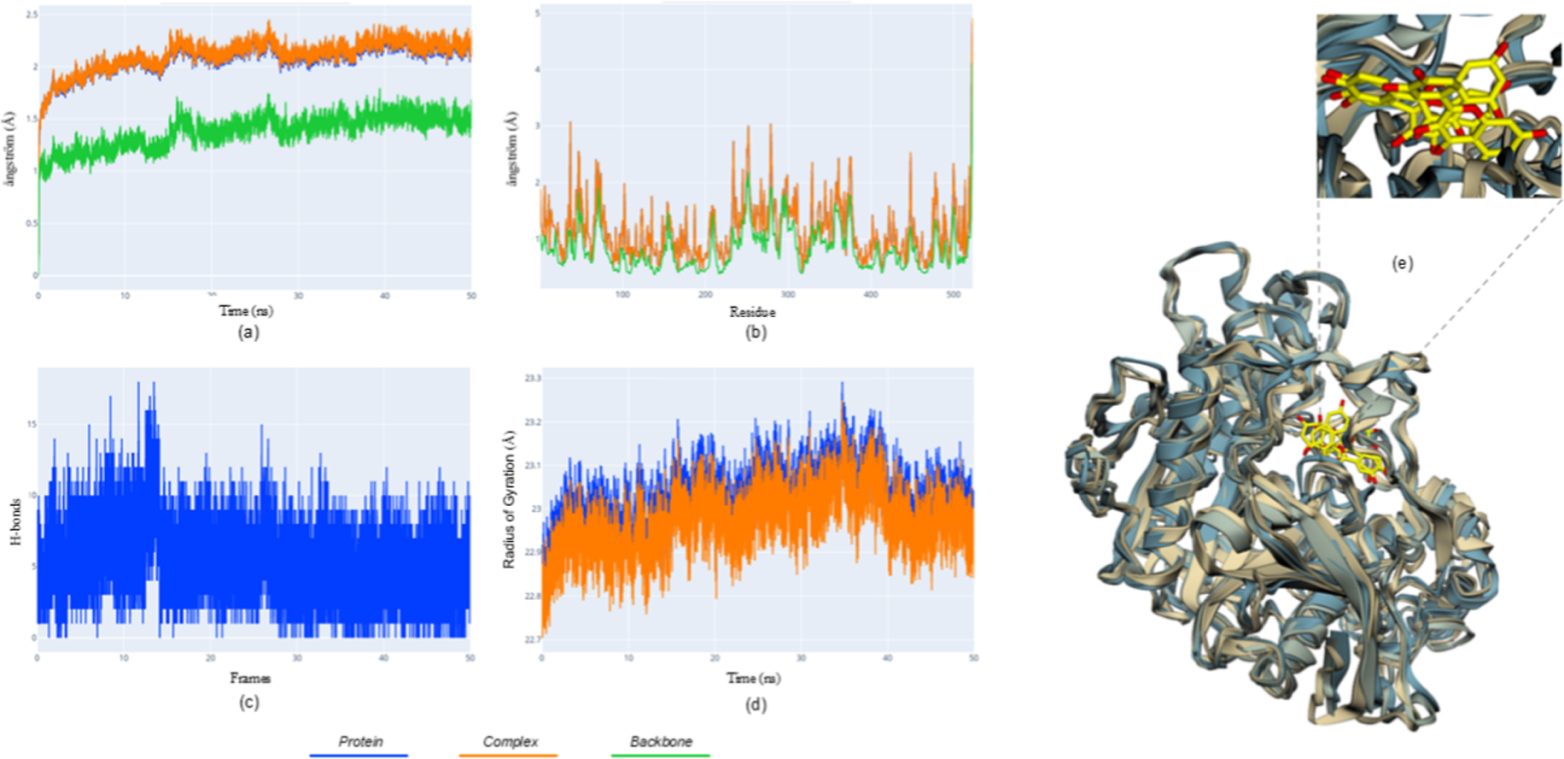
Molecular dynamics results for the BChE-quercetin complex. (a) rmsd; (b) rmsf; (c) hydrogen bonds; (d) Rg; (e) superposed frames showing quercetin stably positioned within the catalytic pocket.

**Figure 7:**
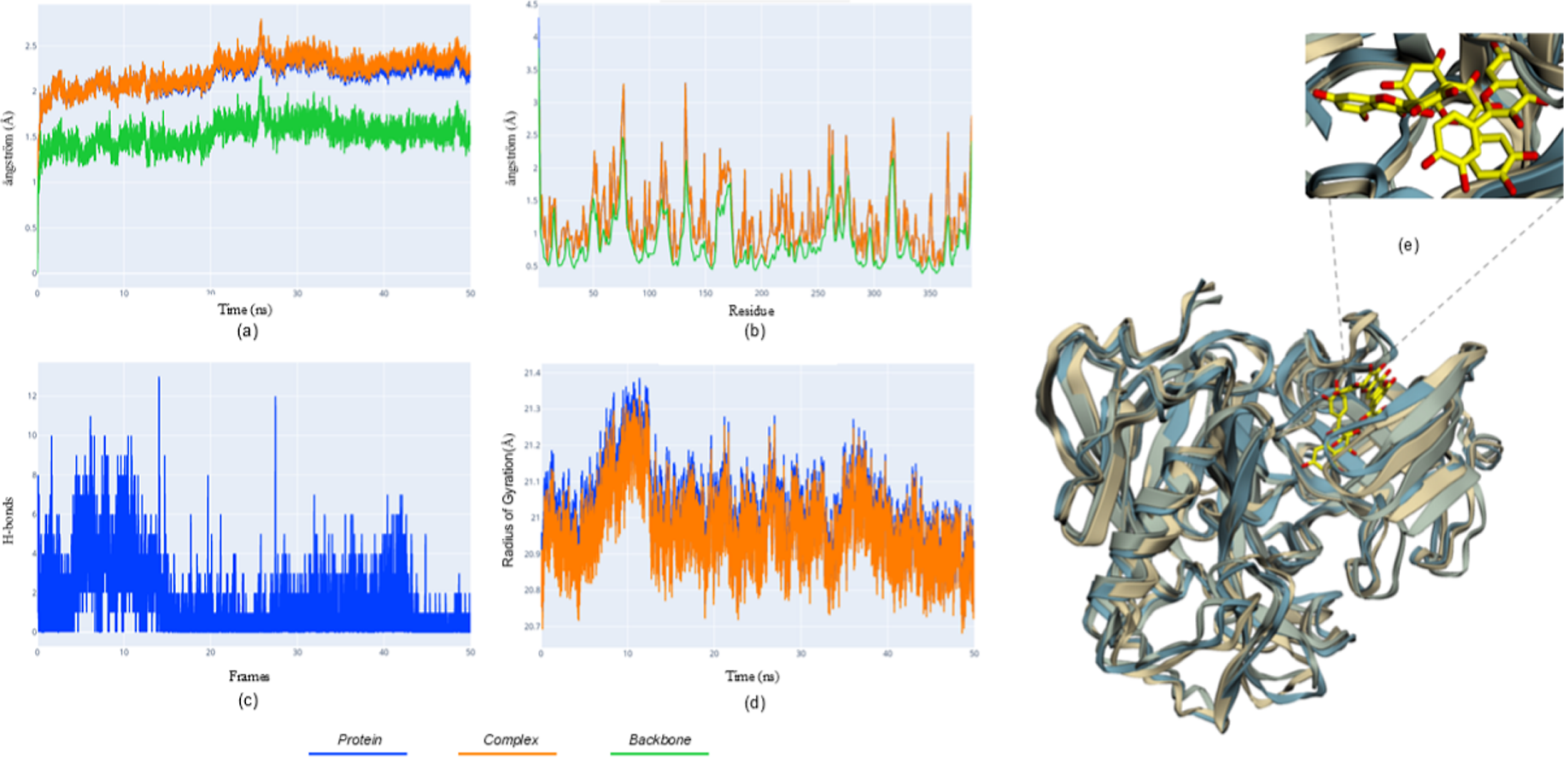
Molecular dynamics results for the BACE1-quercetin complex. (a) rmsd; (b) rmsf; (c) hydrogen bonds; (d) Rg; (e) superposed structures illustrating stable ligand retention near the flexible flap region.

For the AChE-quercetin complex (Figure 5), the rmsd profile showed transient deviations, which in some trajectories became more pronounced at later simulation times, indicating ligand rearrangement within the binding pocket. Across independent simulations, both stable and more dynamic conformational regimes were observed, suggesting that the system can access multiple energetically accessible states depending on initial conditions.

Rmsf values remained low overall, with increased mobility primarily restricted to peripheral loops, indicating preservation of the catalytic core. Hydrogen-bond interactions fluctuated throughout the simulations but were consistently maintained, supporting persistent ligand engagement. The Rg profile remained largely stable, confirming preservation of global compactness. Structural superposition revealed a progressive migration of quercetin toward the peripheral anionic site (PAS), where π–π stacking interactions with aromatic residues, particularly Trp278 and Tyr333, became prominent.

Collectively, these observations indicate a dynamically stable binding mode characterized by conformational adaptability rather than a single rigid binding state.

The BChE-quercetin complex (Figure 6) exhibited a predominantly stable dynamic profile across simulations. Rmsd values plateaued early and remained consistent in most trajectories, indicating preservation of the ligand–protein arrangement following equilibration. Rmsf analysis revealed limited fluctuations, primarily confined to peripheral regions that do not participate directly in catalysis, supporting the structural stability of the active site.

Hydrogen-bond counts remained relatively consistent across simulations, and the Rg profile showed minimal variation, confirming maintenance of global compactness. Structural superposition further demonstrated that quercetin remained consistently positioned within the catalytic pocket, sustaining interactions with key residues.

Although a late deviation was observed in one replicate, the overall behavior indicates a robust and reproducible binding mode for the BChE-quercetin complex.

For the BACE1-quercetin complex (Figure 7), rmsd profiles reached apparent stabilization after the equilibration phase; however, greater variability was observed across independent simulations. Notably, all trajectories consistently exhibited fluctuations in hydrogen-bond interactions and dynamic rearrangements within the binding site.

Rmsf analysis revealed pronounced flexibility in the flap region, a hallmark of BACE1 dynamics, which contributes to ligand accommodation. Hydrogen-bond counts showed a tendency toward fluctuation and, in some trajectories, gradual reduction over time, suggesting a less persistent interaction profile compared to the cholinesterases. The Rg profiles displayed moderate oscillations consistent with the intrinsic flexibility of the system.

Structural superposition confirmed a dynamic mode of ligand association, in which quercetin remains engaged within the active-site cleft but undergoes continuous reorientation. This behavior reflects a more transient binding mode driven by the conformational plasticity of BACE1.

Across all systems, the simulations reached equilibrium within the analyzed time frame, and the overall protein folds were preserved. Importantly, the comparative analysis of independent trajectories demonstrates that, despite stochastic differences at the level of individual simulations, the key dynamic features of each system are reproducible. These results support the reliability of the MD-derived insights and provide a consistent structural basis for subsequent energetic and binding-affinity analyses.

Computational approaches demonstrated that quercetin exhibits a multitarget inhibitory profile against AChE, BChE, and BACE1. Docking and MD simulations revealed distinct binding behaviors across the targets, with a predominantly stable interaction profile for BChE, a dynamically adaptable binding mode for AChE, and a more transient interaction pattern for BACE1.

The strongest binding affinities were observed for BChE, followed by AChE and BACE1, consistent with prior experimental evidence indicating concentration-dependent inhibition of cholinesterases.^28^ Hydrogen-bonding interactions between quercetin’s hydroxyl groups and residues such as Trp278, Phe330, Tyr333, and Tyr121 (AChE), as well as Trp79, Gly113, and His433 (BChE), contribute to ligand stabilization within catalytic and peripheral sites, supporting a dual modulatory mechanism involving catalytic inhibition and allosteric interference.^29^

In BACE1, quercetin engages residues such as Asp36, Tyr75, and Trp191, consistent with the interactions observed in docking, which involve the catalytic environment and the flap region; however, the interaction remains dynamically labile, reflecting the intrinsic flexibility of this region.^30^ These findings align with experimental reports describing quercetin-mediated modulation of cholinergic activity and amyloidogenic pathways, with IC_50_ values in the micromolar range.^30,31^

Together, these results highlight the importance of considering dynamic variability in protein–ligand interactions and support the potential of quercetin as a multitarget compound acting through distinct binding regimes across different enzymes. Its neuroprotective actions are multifaceted, including scavenging reactive oxygen species, attenuating neuroinflammatory responses, and modulating key AD-related mechanisms such as cholinergic dysfunction, BACE1-mediated Aβ production, and tau hyperphosphorylation.^32,33^

### In Vivo Experimental Model

Quantitative histological analyses demonstrated differences in neuronal density across the experimental groups in both the dentate gyrus (DG) and the subventricular zone (SVZ) (*n* = 8 animals per group). For histological quantification, multiple micrographs were obtained from each animal in the analyzed regions, and the mean value per animal was used for statistical analysis. Statistical comparisons among groups were performed using one-way analysis of variance (ANOVA), followed by appropriate post hoc testing when significant differences were detected. Effect size estimates were also considered to aid in the interpretation of the magnitude of group differences.

In the DG (*p* = 0.0276), rats in the control group showed a marked reduction in neuronal density compared with the Sham group, consistent with hippocampal neuronal loss following AD induction. In contrast, quercetin-treated animals (C1 group) exhibited neuronal densities comparable to those of the Sham group, indicating a protective effect on hippocampal integrity (Figure 8).

**Figure 8:**
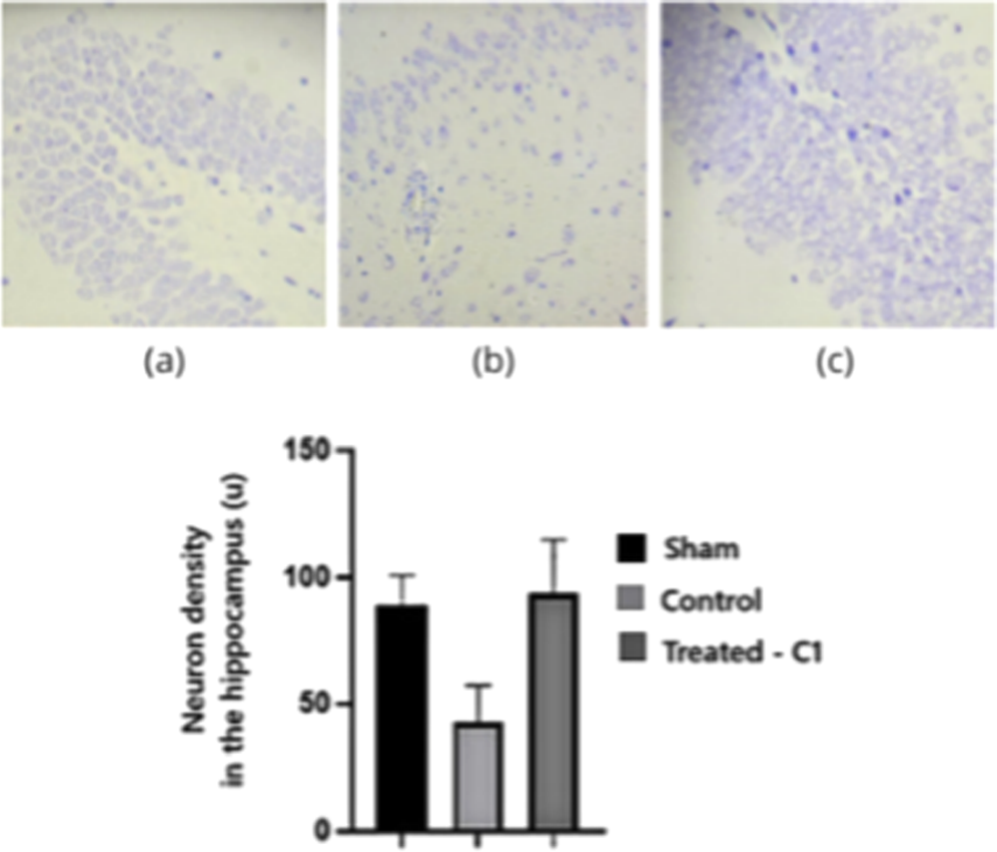
Neuronal density in the dentate gyrus (DG) of rat hippocampi stained with Nissl (Cresyl Violet). (a) Sham group; (b) control group; (c) quercetin-treated group (C1). Magnification: 100×. Statistical comparisons were performed using one-way ANOVA (*p* < 0.05).

A similar pattern was observed in the SVZ (*p* < 0.05), where neuronal density was substantially reduced in the control group, whereas animals receiving quercetin displayed values nearly identical to those of the Sham group. This recovery in both neurogenic regions suggests a neuroprotective effect of quercetin in the in vivo model. The quantitative measurements for both the DG and SVZ are presented in Table 3.

**Table 3:** Neuronal Density in the DG and SVZ across Experimental Groups^a^

region	group	mean	SD
	Sham	89.08	11.84
DG	control	42.90	14.49
	treated-C1	95.58	15.57
	Sham	41.78	21.40
SVZ	control	16.58	11.80
	treated-C1	41.54	21.17

a Data are presented as mean ± SD (*n* = 8 animals per group). SD = standard deviation.

Astrocytic density analysis revealed apparent group-dependent differences in both neurogenic regions examined. In the DG, GFAP immunoreactivity was significantly higher in the control group than in the treated-C1 animals (*p* = 0.0353), indicating that AD induction elicited a marked astroglial response that was attenuated by quercetin administration. A similar pattern was observed in the SVZ, where GFAP labeling differed significantly among the groups (*p* = 0.0244). Animals receiving quercetin exhibited astrocytic densities comparable to those of the Sham group, whereas untreated AD-induced rats showed increased GFAP expression, consistent with elevated glial reactivity (Figure 9).

**Figure 9:**
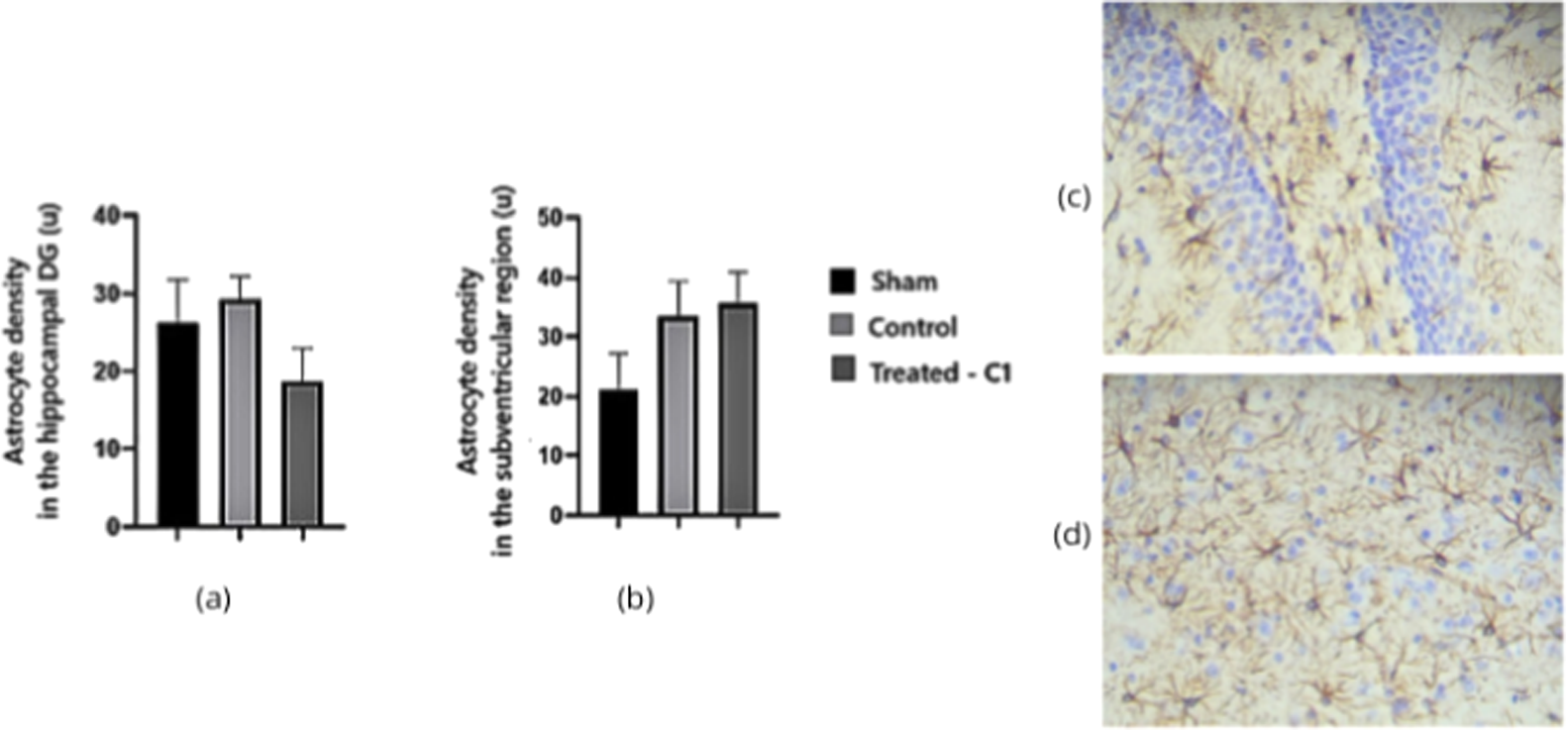
Astrocyte density in the DG (a) and SVZ (b) of rat brains assessed by GFAP immunohistochemistry. (c) Representative GFAP immunolabeling in the DG of the Sham group; (d) GFAP immunostaining in the SVZ of the quercetin-treated group (C1). Magnifications: 100× and 200×. Statistical comparisons were performed using one-way ANOVA (*p* < 0.05).

Quantitative analysis of astrocytic lesion areas revealed highly significant differences among groups in both the hippocampal DG and the SVZ (*p* < 0.001). As shown in Figure 10 and summarized in Table 4, animals in the control group exhibited markedly enlarged lesion areas in both regions, reflecting pronounced astrocytic reactivity following AD induction. In contrast, quercetin-treated animals (C1) exhibited substantially smaller lesion areas than untreated AD rats, approaching the values observed in the Sham group. These findings indicate that quercetin attenuated astroglial damage in both the DG and the SVZ.

**Figure 10:**
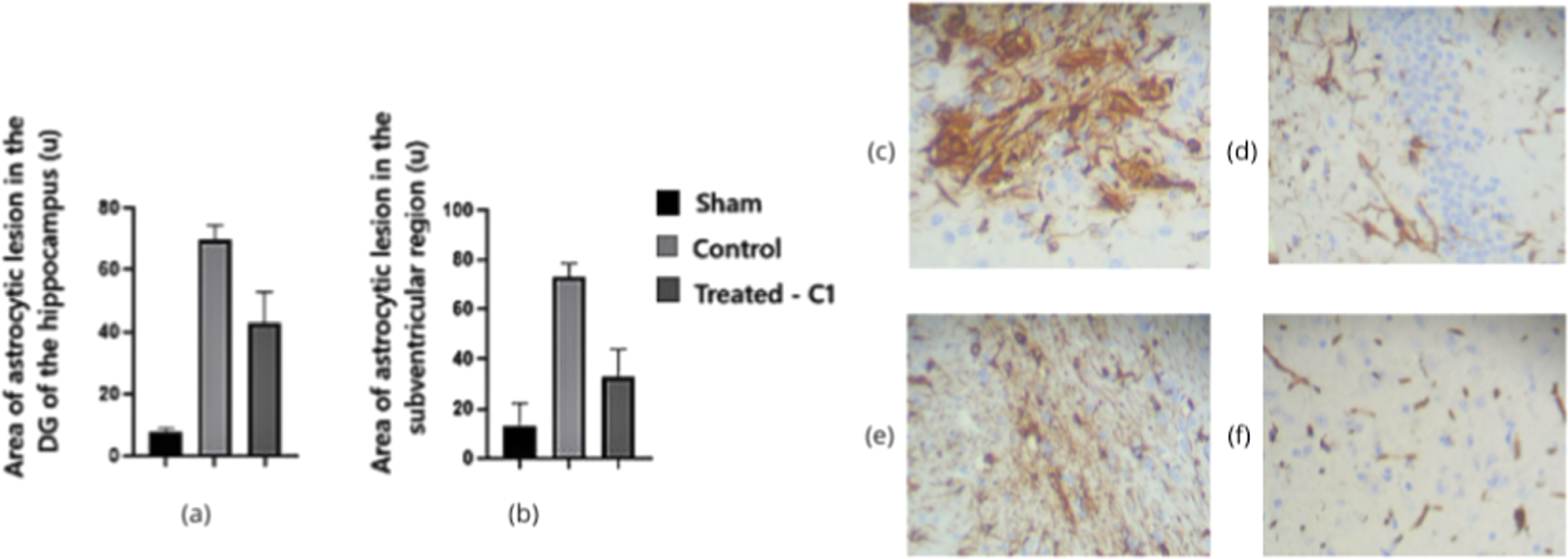
Areas of astrocytic lesion evaluated by vimentin immunohistochemistry in the DG (a) and SVZ (b) regions. (c) DG section from the control group; (d) DG from the quercetin-treated group (C1); (e) SVZ from the control group; (f) SVZ from the quercetin-treated group (C1). Magnification: 100×. Statistical comparisons were performed using one-way ANOVA (*p* < 0.05).

**Table 4:** Descriptive Statistics for Astrocytic Lesion Areas in the DG and SVZ^a^

region	group	mean	SD
	Sham	7.75	1.352
DG	control	69.74	4.573
	treated-C1	42.74	10.02
	Sham	13.20	8.832
SVZ	control	73.14	5.392
	treated-C1	33.02	11.02

a Data are presented as mean ± SD (*n* = 8 animals per group). SD = standard deviation.

The in vivo results obtained in the present study reinforce and extend these computational predictions. Histological analyses revealed that quercetin-treated rats exhibited significantly higher neuronal density in both the DG and SVZ compared with untreated AD animals, approaching those of the Sham group. This neuroprotective effect may be related to previously reported actions of quercetin on cholinergic signaling and neuronal survival pathways.^34,35^ Given that the hippocampus is critically involved in memory encoding and is especially vulnerable to AD-related neurodegeneration,^36^ the preservation of hippocampal neurons strongly suggests the activation of pro-survival and neurogenic pathways. These may involve muscarinic and nicotinic receptor signaling, which have been associated with neuronal differentiation and synaptic maintenance.^37,38^

In parallel with its neurogenic effects, quercetin markedly modulated astrocytic responses, as evidenced by increased GFAP immunoreactivity and reduced astrocytic lesion areas in both the hippocampal and subventricular regions. Astrocytes exert a dual influence in AD pathophysiology: while reactive astrocytosis accompanies neuroinflammation, functionally supportive astrocytes facilitate Aβ clearance and secrete neurotrophic factors such as BDNF and NGF.^39,40^ The increased astrocytic density observed in quercetin-treated animals may therefore reflect an adaptive, protective shift rather than pathological activation. This interpretation is supported by the concomitant reduction in vimentin-marked astrocytic lesions, indicating attenuation of glial stress and partial restoration of tissue homeostasis. Liddelow et al. demonstrated that polyphenols similar to quercetin can reprogram astrocytic phenotypes toward neuroprotection, which closely aligns with the present findings.^41^

The convergence of in silico and in vivo data provides a mechanistic framework to interpret the observed neuroprotective phenotype. Quercetin’s multifunctional chemical scaffold, composed of hydroxylated aromatic rings and a conjugated chromone core, enables interactions with diverse enzyme families while potentially modulating oxidative and inflammatory pathways. Its well-documented antioxidant properties may help counteract excessive ROS generation associated with lipid peroxidation, protein damage, and DNA instability in AD.^42^ Moreover, by potentially modulating BACE1-mediated amyloidogenesis and cholinergic signaling, quercetin acts on two fundamental pathological axes of the disease.^28,43^ In alignment with these mechanistic pathways, quercetin-treated animals displayed reduced GFAP and vimentin reactivity, decreased astrocytic lesion areas, and increased neuronal and astrocytic densities in neurogenic niches, supporting the translational relevance of the computational predictions.

Building on this mechanistic framework, the present study provides evidence that quercetin acts as a potent multitarget modulator capable of mitigating key pathological processes associated with AD. The integration of in silico and in vivo approaches suggested that quercetin can establish stable interactions with AChE, BChE, and BACE1, three enzymes critically involved in cholinergic dysfunction and amyloidogenesis. Molecular docking and MD simulations revealed that these interactions are mediated by π–π stacking, hydrogen bonding, and van der Waals forces involving catalytically relevant residues, suggesting favorable and system-dependent dynamic stability. Consistently, in vivo administration of quercetin in the STZ-induced rat model restored neuronal integrity and attenuated astrocytic reactivity, as reflected by increased neuronal density and reduced GFAP and vimentin expression, indicating the modulation of neuroinflammatory cascades alongside direct neuroprotective effects.

Overall, the convergence of computational predictions with experimental validation supports the potential of quercetin as a multitarget therapeutic scaffold for AD and highlights the value of an integrated computational-experimental strategy for advancing the rational design of multifunctional therapeutics targeting complex neurodegenerative disorders.

## Methods

### Computational Methods

The neuroprotective potential of quercetin in AD was investigated through an integrated design combining in silico analyses and in vivo experiments. These two components were developed in parallel to allow molecular-level observations to be directly related to biological and histological outcomes.

The in silico work focused on the interaction of quercetin with three enzymes closely linked to AD pathophysiology, AChE, BChE, and BACE1. To explore these interactions, we employed structure-based virtual screening, consensus docking, and MD simulations. These methods provided complementary information regarding binding affinity, key stabilizing interactions, and the conformational behavior of the protein–ligand complexes, offering a mechanistic basis for assessing quercetin’s potential inhibitory activity.

Compounds were retrieved from the ChEMBL database v34.0 ^16^ based on reported activity toward the selected enzymatic targets AChE, BChE, and BACE1. The initial collection underwent a structured refinement process to ensure chemical consistency and biological relevance. Duplicate entries were removed, and only compounds supported by experimental data from biologically meaningful organisms, particularly *H. sapiens*, were retained. Molecules displaying unfavorable physicochemical characteristics, such as excessive molecular weight or low predicted solubility, were excluded, as were entries with incomplete or inconsistent structural descriptors. These filtering steps were performed using DataWarrior v6.04,^44^ complemented by similarity analyses conducted with ChEMBL’s cheminformatics tools in Python.

Once curated, the data set was examined for chemical patterns and recurring structural motifs that might underlie activity across the target enzymes. To further interpret these findings, a comprehensive computational workflow was established encompassing receptor preparation, binding-site characterization, consensus docking, and MD simulations, forming a coherent and reproducible framework for analyzing how quercetin and structurally related molecules engage the selected targets.

Crystallographic complexes corresponding to the study’s enzymes were obtained from PDB.^15^ Receptor preparation included removal of water and solvent molecules, addition of hydrogens, and assignment of Gasteiger partial charges using UCSF Chimera v1.18.^45,46^ Ligand structures were parametrized in Chimera using AM1-BCC partial charges.^46,47^

Molecular docking was conducted using a consensus-based approach^48^ integrating AutoDock Vina^25^ and DOCK6.^26^ Validation of the docking protocol was performed through redocking of the cocrystallized ligands extracted from each PDB structure. The predicted poses were compared to the experimental coordinates by calculating the root-mean-square deviation (rmsd)^49^ using PyMOL v3.^50^ rmsd values ≤2.0 Å were considered successful reproductions of native binding modes, confirming the reliability of the docking parameters. For the full ligand set, top-ranked AutoDock Vina^25^ poses were subsequently reprocessed in DOCK6,^26^ and the final consensus binding score was calculated as the arithmetic mean of the two outputs, providing a more stable and reliable estimate of binding affinity.

The hit compounds identified through consensus docking were subsequently subjected to MD simulations to evaluate the stability and persistence of their interactions with the target enzymes. MD simulations were performed using the OpenMM package v8.1.1.^51^ Protein parameters were assigned using the AMBER ff14SB force field, while ligand parameters were generated with the Open Force Field (OpenFF) small-molecule force field. Each protein–ligand complex was solvated in an explicit TIP3P solvent model within an orthorhombic box extending 10 Å beyond the solute. Sodium and chloride ions were added to achieve a final concentration of 0.15 M, reproducing physiological ionic strength.

Following energy minimization, the systems underwent a 100,000-step equilibration phase (200 ps), after which 50 ns production simulations were performed under isothermal–isobaric (*NPT*) conditions using a 2.0 fs integration time step. Temperature was maintained at 310 K through a Langevin integrator with a friction coefficient of 1.0 ps^–1^, and pressure was controlled at 1 atm using a Monte Carlo barostat applied every 25 steps. All covalent bonds involving hydrogen atoms were constrained. Long-range electrostatic interactions were treated using the particle-mesh Ewald (PME) method with a real-space cutoff of 10 Å. Trajectory coordinates and checkpoint files were recorded every 1000 steps. Simulations were executed on a CUDA-enabled NVIDIA GPU platform.

To evaluate the robustness of the MD results, three independent simulations were performed for each protein–ligand complex. In addition to the initial trajectory, two replicate simulations were carried out using different random seeds (37 and 73) to generate independent initial velocity distributions. All simulations were conducted under identical conditions.

The dynamic behavior of the systems was characterized using rmsd, rmsf, Rg, and the number of protein–ligand hydrogen bonds. The results were analyzed comparatively across independent trajectories to assess the consistency of the observed dynamic patterns.

### Experimental Methods

Twenty-four male Wistar rats (*Rattus norvegicus*, var. *albinus*), aged 40 days and weighing between 250 and 450 g, were used in this study. The animals were obtained from the Central Animal Facility of the Federal University of São João del-Rei (UFSJ), and the experimental project was submitted to the university’s Ethics Committee Involving the Use of Animals (CEUA).^52^ The animals were maintained under standardized husbandry conditions to ensure experimental reproducibility.

Rats were housed in polypropylene cages under a 12 h reversed light/dark cycle with standardized artificial lighting. The room temperature was maintained between 21 and 22 °C, and the relative humidity between 60% and 70%. Throughout the experiment, animals had ad libitum access to filtered water and balanced commercial chow, ensuring adequate nutritional conditions for the development of the experimental model.

The compound showing the highest binding affinity in the vHTS analysis was administered orally at a dose of 30 mg/kg via gavage, allowing precise dosing and minimizing variability in bioavailability. The treatment regimen lasted 5 weeks, with administration delivered five times per week, beginning in the postoperative period following AD induction or Sham procedures. All treatments were conducted under strict monitoring to minimize animal stress and preserve the integrity of the experimental model.^53^

AD-like model was induced by stereotaxic ICV administration of streptozotocin (STZ; Sigma-Aldrich Brasil Ltd.a.) at a dose of 10 μg in 2 μL. To access the target region, a cranial trephination was performed using a spherical dental bur attached to a low-speed motor, creating an opening approximately 1.5 mm in diameter between Lambda and Bregma.

STZ was injected according to stereotaxic coordinates referenced from Bregma, AP (anteroposterior): 0.8 mm, ML (mediolateral): 1.4 mm, and DV (dorsoventral): 3.4 mm, following the Paxinos and Watson Atlas.^54^ Microinjections were performed with a 5 μL Hamilton syringe, which was left in place for 5 min before gradual withdrawal to minimize reflux.

After STZ administration, the scalp was sutured, and a topical antibiotic (Dermolene) and an oral ketoprofen (Medley; 20 mg/mL, 1 drop) were administered to prevent infection and ensure postoperative analgesia.

After completion of the experimental protocol, animals were euthanized in accordance with ethical guidelines, and the brains were removed for histological analysis. Brain tissue was fixed in 10% buffered formalin for 24 h and subsequently stored in 70% ethanol until processing. Samples were embedded in paraffin and sectioned sagittally at an approximate thickness of 1 mm.^55^

To assess neuronal integrity and tissue organization, histological sections were stained with Nissl. The method consists of immersing the sections in a cresyl violet solution (Sigma-Aldrich Brasil Ltd.a.), which highlights the neuronal cytoplasm and Nissl bodies, structures essential for protein synthesis. This staining served as an indicator of neuronal viability, as chromatolysis, characterized by the disappearance of Nissl bodies, is a histological marker of neuronal injury.^19^

The histochemical procedure followed the dehydration and clearing steps described by Vasconcelos et al.^55^ Sections were immersed for 5 min each in xylene I, xylene II, absolute ethanol (100%), 95% ethanol, and 70% ethanol, then incubated in 0.5% cresyl violet for 30 min. After staining, sections underwent progressive dehydration, clearing in xylene, and permanent mounting. Microscopic analyses focused on the SVZ and the DG of the hippocampus.

Astrocytic responses were assessed by immunostaining for GFAP and vimentin (VM), well-established biomarkers of astrocyte reactivity and glial injury. The expression of these markers was investigated to determine the potential neuroprotective effects of the tested compounds in the experimental model. Detection was performed using specific primary antibodies: polyclonal anti-GFAP (Rabbit anticow, Code Z0334, Dako) and monoclonal anti-VIM (Mouse antiswine, Code M0725, Dako).^56^

Histological sections were washed in phosphate-buffered saline (PBS) and incubated with a biotinylated secondary antibody (Dako LSAB2-HRP, Dako Cytomation) for 2 h, followed by incubation with a streptavidin-peroxidase conjugate for 45 min. Immunolabeling was visualized using the 3,3′-diaminobenzidine (DAB; Sigma-Aldrich) chromogen at a 1:2 ratio, followed by nuclear counterstaining with hematoxylin.

This procedure enabled qualitative and quantitative evaluation of astrocytic distribution, reactivity, and histopathological features in the treated brain tissue,^57^ using brightfield microscopy.

Histomorphometric analysis of the subgranular zone of the DG and the SVZ was performed using ImageJ software.^58^ Slide images were acquired with a photonic microscope-mounted digital camera, and neuronal counts were quantified using ImageJ’s standardized measurement tools.

The data were analyzed using GraphPad Prism 9.0.^59^ Group comparisons were performed using one-way ANOVA, followed, when appropriate, by Tukey’s posthoc test. The normality of the data was verified by the Shapiro–Wilk test. Results are expressed as mean ± standard deviation (SD), and statistical significance was set at *p* < 0.05.

## Supplementary Material



## Footnotes

a Aβ_1–42_ refers to the amyloid-β peptide comprising amino acid residues 1 to 42 derived from the APP, representing the most aggregation-prone and neurotoxic isoform.

## Notes

Figures S1–S6, showing molecular dynamics results (rmsd, rmsf, protein–ligand hydrogen bonds, and radius of gyration) for AChE-, BChE-, and BACE1-quercetin complexes from independent simulation replicates (seeds 37 and 73) (PDF)

## Abbreviations

AD: Alzheimer’s disease
MD: molecular dynamics
AChE: acetylcholinesterase
BChE: butyrylcholinesterase
BACE1: β-secretase 1
vHTS: virtual high-throughput screening
rmsd: root-mean-square deviation
rmsf: root-mean-square fluctuation
Rg: radius of gyration
STZ: streptozotocin
GFAP: glial fibrillary acidic protein
ICV: intracerebroventricular
DG: dentate gyrus
SVZ: subventricular zone
APP: amyloid precursor protein
