# Supplementary material for: Integrative In Silico and In Vivo Evidence of Quercetin as a Multitarget Neuroprotective Agent in Alzheimer’s Disease
Source: ACS Omega. 2026 Apr 29;11(18):27115–27. doi: 10.1021/acsomega.6c00778 (PMC13177005; doi:10.1021/acsomega.6c00778)
Supplement: Supplementary file 1 [file ao6c00778_si_001.pdf]

Integrative *In Silico* and *In Vivo* Evidence of Quercetin as a Multitarget Neuroprotective Agent in Alzheimer's Disease

Thiago Malverde de Oliveira[1]; Lucas Diego Pereira Bento[1]; Isabela Santos de Melo Wiermann[2]; Bianca de Souza Fonseca[2]; Guilherme Saraiva Tsui[3]; Carolina Jayne Pereira de Jesus[1]; Raphaela Oliveira Sales[3]; Mateus Antonio Pereira Prado[3]; Mateus Silva de Castro[3]; João Pedro Reis Moura[3]; Ellen Nunes Gomes[3]; Lavinia Brito Bastos[3]; Izabela Cristina Lima Orsine[3]; Lucas de Souza Esteves[3]; Maria Clara Silva Soares[3]; Daniel Luciano Falkoski[2]; Michel Pires da Silva[2,4]; Tiago Alves de Oliveira[2,4]; Alisson Marques da Silva[4]; Eduardo Habib Bechelane Maia[4]; Franco Henrique Andrade Leite[5]; Marcelo Siqueira Valle[1]; Paulo Batista de Carvalho[6]; Liliane Costa Vanessa Pereira Mendes[3]; Alex Gutterres Taranto[2] ; Laila Cristina Moreira Damázio[3].*

[1]Department of Natural Sciences, Federal University of São João del-Rei, Dom Helvécio Square, 74 - Dom Bosco, 36301-160, Minas Gerais, São João del-Rei-MG.

[2]Department of Biotechnology, Federal University of São João del-Rei, Dom Helvécio Square, 74 - Dom Bosco, 36301-160, Minas Gerais, São João del-Rei-MG.

[3]Department of Medicine, Federal University of São João del-Rei, Dom Helvécio Square, 74 - Dom Bosco, 36301-160, Minas Gerais, São João del-Rei-MG.

[4]Department of Computer Science, State Federal Center for Technological Education of Minas Gerais, R. Alvares de Azevedo, 35503-822, Minas Gerais, Divinópolis-MG.

[5]Department of Health Science, State University of Feira de Santana, Transnordestina Avenue, 44036-900, Bahia, Feira de Santana-BA.

[6]Feik School of Pharmacy, University of the Incarnate Word, San Antonio, Texas, United States.

This Supporting Information contains additional molecular dynamics analyses performed using independent simulation replicates.

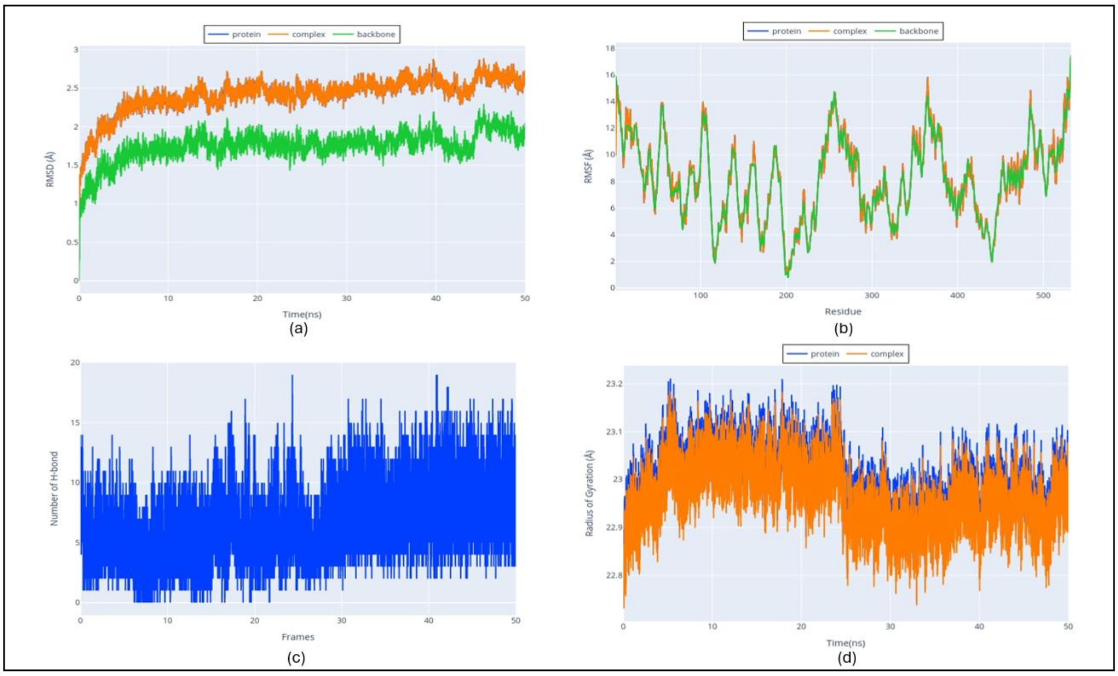

Figure S1. Molecular dynamics results for the AChE-quercetin complex (seed 37). (a) RMSD; (b) RMSF; (c) protein-ligand hydrogen bonds; (d) radius of gyration (Rg).

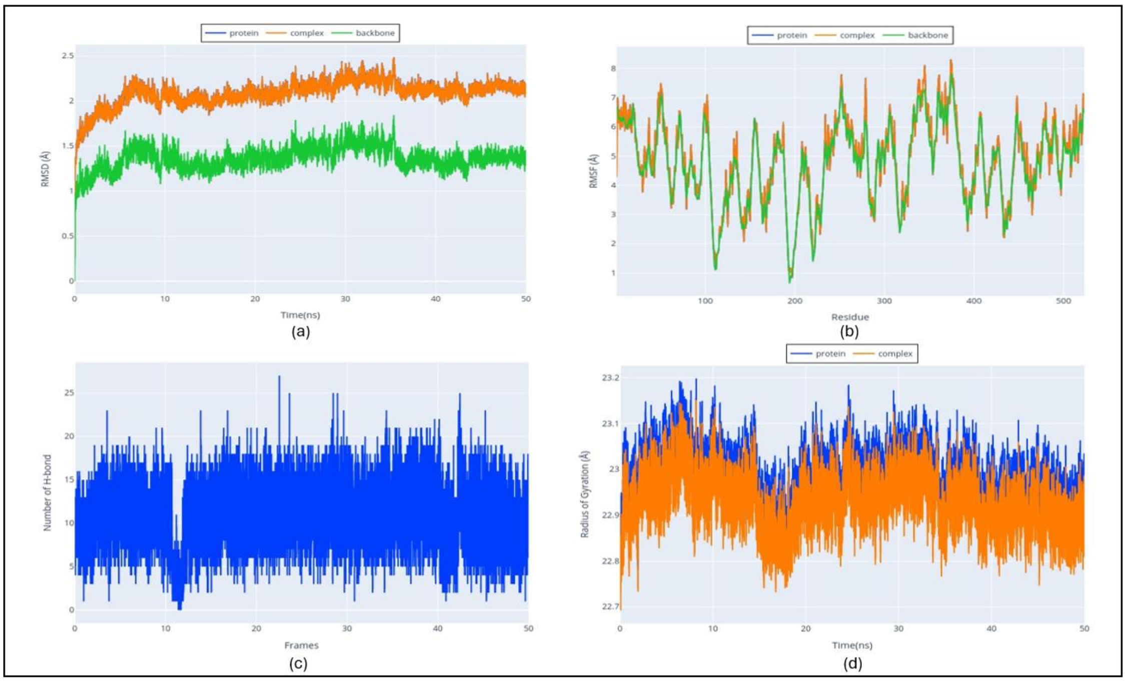

Figure S2. Molecular dynamics results for the BChE-quercetin complex (seed 37). (a) RMSD; (b) RMSF; (c) protein-ligand hydrogen bonds; (d) radius of gyration (Rg).

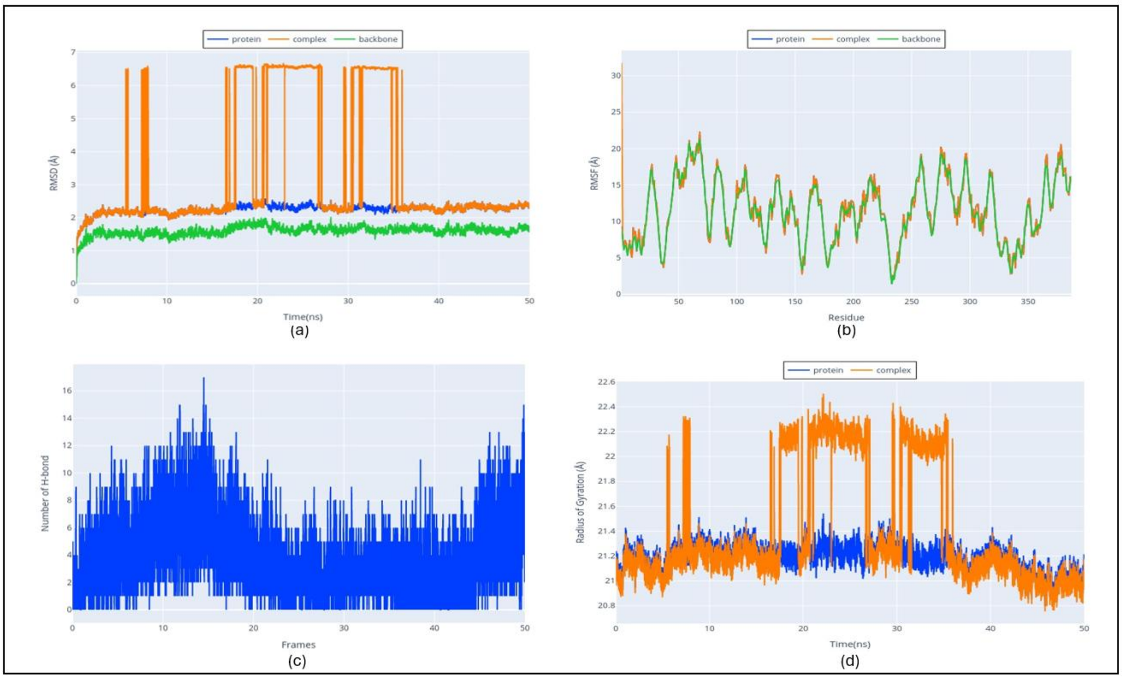

Figure S3. Molecular dynamics results for the BACE1-quercetin complex (seed 37). (a) RMSD; (b) RMSF; (c) protein-ligand hydrogen bonds; (d) radius of gyration (Rg).

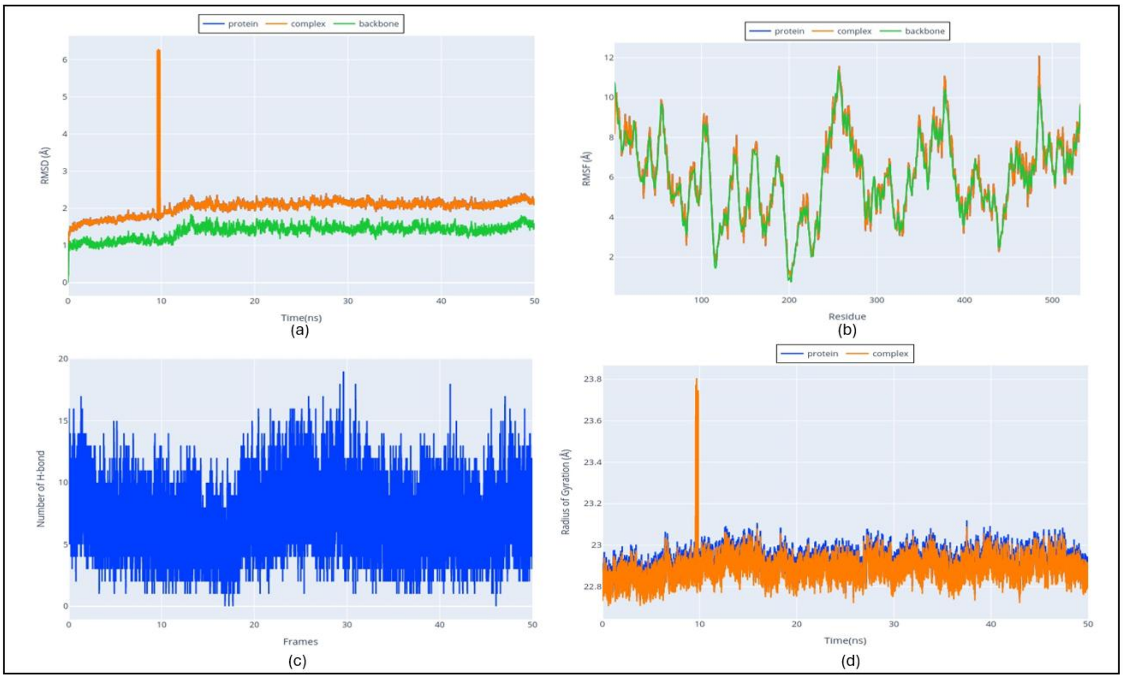

Figure S4. Molecular dynamics results for the AChE-quercetin complex (seed 73). (a) RMSD; (b) RMSF; (c) protein-ligand hydrogen bonds; (d) radius of gyration (Rg).

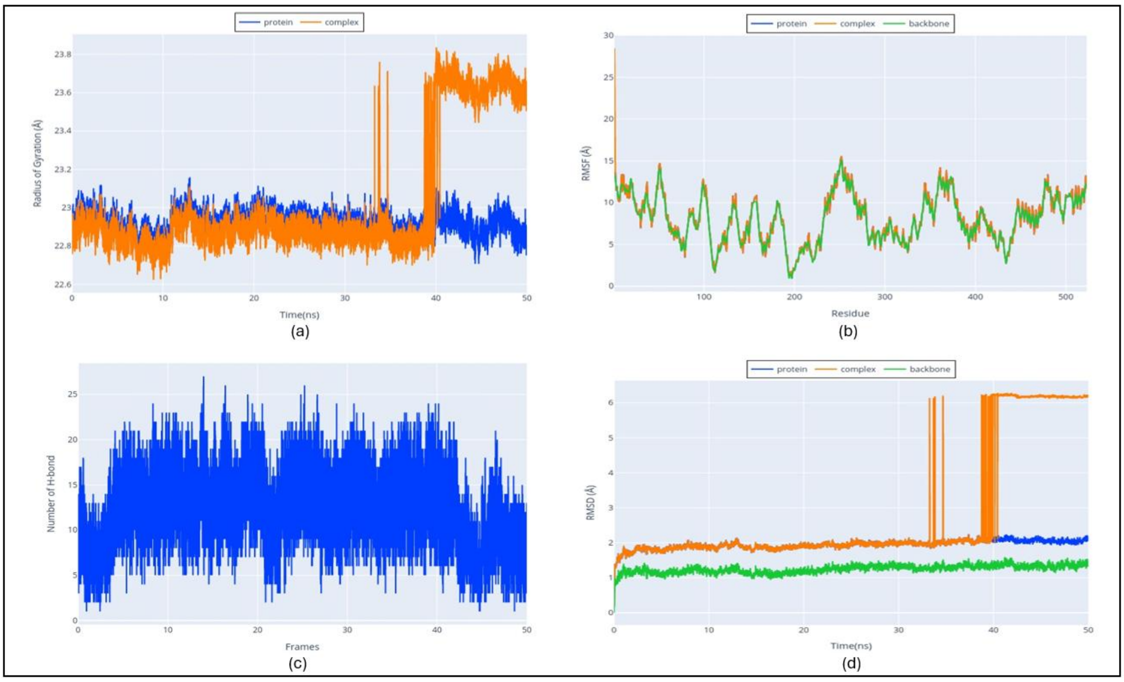

Figure S5. Molecular dynamics results for the BChE-quercetin complex (seed 73). (a) RMSD; (b) RMSF; (c) protein-ligand hydrogen bonds; (d) radius of gyration (Rg).

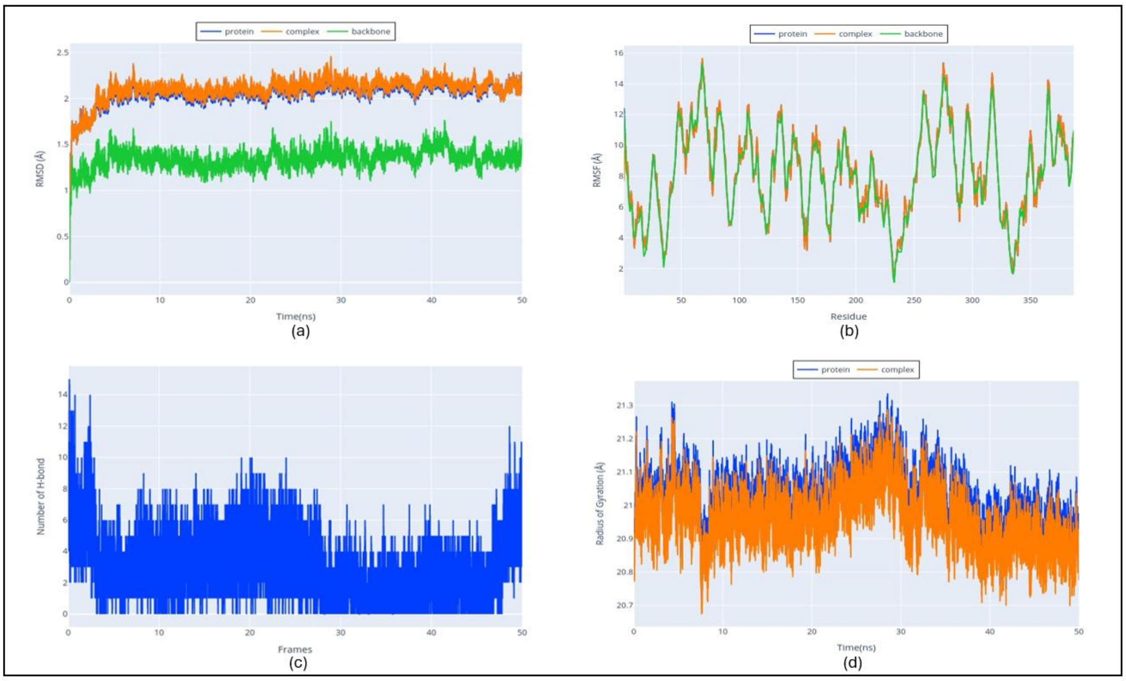

Figure S6. Molecular dynamics results for the BACE1-quercetin complex (seed 73). (a) RMSD; (b) RMSF; (c) protein-ligand hydrogen bonds; (d) radius of gyration (Rg).
